# Evaluation and comparison of synthetic computed tomography algorithms with 3T MRI for prostate radiotherapy: AI‐based versus bulk density method

**DOI:** 10.1002/acm2.14581

**Published:** 2024-11-29

**Authors:** Sakari S. Karhula, Piia Karppinen, Henna Hietala, Juha Nikkinen

**Affiliations:** ^1^ Department of Oncology and Radiotherapy Oulu University Hospital Oulu Finland; ^2^ Research unit of Health Sciences and Technology Oulu University Oulu Finland

**Keywords:** artificial intelligence, clinical commissioning, computed tomography, dosimetry, magnetic resonance imaging, prostate cancer, radiotherapy, synthetic computed tomography

## Abstract

**Purpose:**

Synthetic computed tomography (sCT)‐algorithms, which generate computed tomography images from magnetic resonance imaging data, are becoming part of the clinical radiotherapy workflow. The aim of this retrospective study was to evaluate and compare commercial bulk‐density‐method (BM)‐based and AI (artificial intelligence)‐based‐algorithms using 3T magnetic resonance imaging (MRI) with patient data as part of the local clinical commissioning process.

**Methods:**

44 prostate radiotherapy patients were subjected to MRI and treatment planning CT (TPCT) scans. From the MRI images, sCT images with two different sCT algorithms were generated. The sCT images were evaluated by visual inspection of artifacts. Both sCT methods were compared to TPCT, with Dice similarity score(DSC) of bone and body contours, DVH parameters for CTV, bladder and rectum, and gamma‐analysis. Accuracy for treatment alignment using sCT images was also tested. Various limits were used to define whether the differences between sCT methods to TPCT were clinically relevant (DVH parameters <2%, gamma‐analysis passing rates 90%, 95%, and 98%, and the DSC 0.98 for body and 0.7 for bone).

**Results:**

Our results show that, differences in CTV‐dose coverage values were <2% in most of the patients with both sCT algorithms when compared to reference dose coverage. While AI‐sCT had mean dose coverage difference <0,5% and BM‐sCT <1%. Gamma‐analysis showed that the AI‐sCT mean passing rates were 95.4%, 98.6%, and 99.4% with 1mm1%, 2mm2%, and 3mm3% criteria, respectively. Similarly for BM‐sCT the mean passing rates were 93.4%, 98.2%, and 99.2%. For the treatment alignment accuracy, the mean difference in magnitude of the translational shifts was 1.43 mm for BM‐sCT and 1.57 mm for AI‐sCT. Even though AI‐sCT showed statistically better correspondence to TPCT, the differences were not clinically relevant with any of the limits. Visual evaluation showed artifacts in the AI‐sCT especially in the bowel area and fiducial markers were not generated with either of the sCT algorithms.

**Conclusions:**

In conclusion, sCT‐algorithms were clinically usable on prostate treatments using 3T MR‐only workflow. While AI‐sCT showed better correspondence to TPCT than BM‐sCT, it generated characteristic artifacts. As sCT algorithms perform well, we still recommend testing the sCT‐algorithms with retrospective analyses from patient data prior to implementing sCT into the routine workflow to better understand the specific limitations and capabilities of these algorithms.

## 1 INTRODUCTION

Synthetic computed tomography (sCT) refers to electron density maps generated from magnetic resonance (MRI) images. This provides advantages especially in the clinical workflow of radiotherapy as it could remove the use of computed tomography (CT). In addition to time and cost savings, this also known as MR‐only workflow will be free of errors in co‐registrations between CT and MRI. However, the removal of CT from the radiotherapy workflow means that the possible errors caused by the sCT algorithm generation should be elucidated by clinical commissioning.^1^ The CT provides electron density maps of the patient on which the treatment planning of the radiotherapy relies on, thus the accuracy of the synthetic electron density values should be high to minimize the overall error in the radiotherapy workflow. Furthermore, the sCT images are prone to the same geometrical artifacts as the MRI images they are based on.

There are multiple methods of generating the electron density values in sCT images. The main categories of the sCT categories are bulk density assignment method (BM), atlas‐based techniques, and artificial intelligence (AI)‐based methods. BM‐sCT relies on segmenting the MRI images into different tissue classes (e.g. water, fat, air, bone) and setting predefined density values for each segmented volume.^2^ Atlas‐based methods use a database of CT and MRI images which are co‐registered rigidly and non‐rigidly. Utilization methods of the transformation information from the CT‐MRI atlas co‐registrations vary, but one example is to co‐register target MRI pair‐wise to atlas MRI images and obtain transformation maps. These transformation maps are used to transform the atlas CT images which are then fused together to generate estimation of the electron density map.^2^ The AI‐based sCT as a term includes machine learning and specifically the deep learning (DL) based algorithms used in generating the sCT images. The basics principles in these various methods are the same, the electron density maps are estimated directly from the MRI images. This requires a training phase, in which MRI‐CT image pairs are used to train the algorithm to identify electron density maps from MRI‐intensity images. The algorithm architectures are highly non‐linear utilizing methods that is generator‐only DL methods (i.e. convolutional neural networks) and generative adversarial networks.^3^

High field strength MRIs (3T) provide higher signal to noise ratio and potentially shorter imaging times at the costs of higher susceptibility, increase of chemical shift artifacts and signal voids originating from dielectric effects.^4^ As these artifacts are partially dependent on patient composition, higher field MRI proposes additional sources of uncertainties also for sCT images. The sCT algorithms have been conducted with images scanned with various field strengths (1‐3T) showing promising results.^1^ Previous studies using different sCT methods show high accuracy in brain and pelvic area.^5^, ^6^, ^7^, ^8^, ^9^, ^10^ Also, a comparative study for pelvic area radiotherapy (rectum, anal canal, cervix, or endometrium)^11^ has been conducted between different “in‐house” sCT algorithms. Still, these studies have utilized algorithms which are not commercially available. Recently published study was first to compare four different commercially available sCT algorithms, based on 1.5T MRI images, used for the prostate and brain areas.^12^ However, similar comparison of commercial algorithms with higher MRI fields remains to be conducted. These type of comparative studies evaluating multiple sCT algorithms would help researchers and algorithm manufacturers to focus the development on the sCT generation methods which show most potential for the clinical use.

As the sCT images provide the information to the dose calculation systems instead of the CT‐data, the recommendations proposed by the American Association of Physicists in Medicine (AAPM)^13^ and the European Federation of Organisations from Medical Physics (EFOMP)^14^ regarding the validity of dose calculations should be applicable to the dose calculations based on the sCT‐data. Thus, it is recommended for medical physicists to evaluate the uncertainties and limitations these new algorithms propose, prior to utilization of the algorithms in the treatment workflow. For this study, we evaluated and compared two 3T MRI‐only workflows, using different sCT algorithms, against a standard CT‐based workflow. The evaluated sCT algorithms were a commercial BM‐sCT algorithm included in the medical product syngo.via RT Image Suite VB50 (Siemens Healthineers) and a newer AI‐sCT algorithm by Siemens Healthineers. For the latter AI algorithm, we used a software prototype provided by Siemens Healthineers. The AI‐based Synthetic CT algorithm evaluated in this study has meanwhile been released as part of the medical product syngo.via RT Image Suite VB60. The aim of this study was to compare the performance of these two algorithms, based on 3T MR images, and to test their clinical usability for prostate radiotherapy.

## 2 MATERIALS AND METHODS

### 2.1 Patient selection and radiotherapy treatment information

A total of 44 patients undergoing prostate radiotherapy, were included in this retrospective study. Approval for this non‐interventional, registry‐based study was obtained from the Northern Ostrobothnia Hospital District. The average age of the patients was 71,4 (± 5,8) years. In Table 1, dose prescriptions, radiotherapy targets, and volumes are presented.

**TABLE 1:** Patient and radiotherapy treatment overview.

Prescribed dose	Treatment type	Treatment targets	Amount of patients
60 Gy (3.0 Gy × 20)	^a^SIB	Prostate & seminal vesicles	18
76 Gy (2.0 Gy × 38)^b^	Sequential boost	Prostate, seminal vesicles, and lymph nodes	14
66 Gy (2.0 Gy × 33)^c^	Sequential boost	Prostate bed and lymph nodes	2
76 Gy (2.0 Gy × 38)^d^	SIB	Prostate	9
45 Gy (1.8 Gy × 25)	No boost	Prostate, seminal vesicles, and lymph nodes	1

^a^ SIB = Simultaneous‐Integrated boost. The lower dose areas to the prostate margin and seminal vesicles were 57.6 Gy (2.88 Gy × 20) and 48 Gy (2.4 Gy × 20). Only the highest prescribed dose (60 Gy) and dose volume (prostate) were included in this study.

^b^ The lower dose areas to seminal vesicles and lymph nodes were 66 Gy (2.0 Gy × 33) and 46 Gy (2.0 Gy  × 23). Only the highest prescribed dose (76 Gy) and dose volume (prostate) were included in this study.

^c^ The lower dose area to lymph nodes was 45 Gy (1.8 Gy × 25). Only the highest prescribed dose (66 Gy) and dose volume (prostate bed) were included in this study.

^d^ The lower dose area to prostate margin was 68.4 Gy (1.8 Gy × 38). Only the highest prescribed dose (76 Gy) and dose volume (prostate) were included in this study.

### 2.2 Image acquisition and synthetic CT generation

Patients were subjected to standard prostate radiotherapy protocol, which included pre‐treatment CT and MRI for treatment planning purposes, and cone‐beam computed tomography (CBCT) before each treatment fraction to ensure treatment positioning. Flat couch tops and patient position devices during MRI and CT image acquisitions were used to enable the same patient positioning as in the treatment table. Used imaging parameters are shown in Table 2. Prior imaging 3–4 gold fiducial markers were implanted in patient's prostate for aiding in CT‐MRI image co‐registration (rigid, 6 degrees‐of‐freedom, DOF) and in treatment positioning. In case of post‐op prostate radiotherapy, surgical clips in prostate bed are used for these purposes.

**TABLE 2:** Imaging parameters for MRI, CT, and CBCT.

Modality	Equipment	Parameters
MRI	3T Magnetom Vida, Siemens Healthineers	Resolution: 1.2 mm × 1.2 mm Sequence: 3D Dixon Echo Time: 1.3 ms Repetition Time: 4.3 ms Slice thickness: 1.2 mm Flip Angle: 10˚ Bandwidth: 1093 Hz/pixel
CT	Toshiba Aquilion LB, Canon Medical Systems	Resolution: 0.9 mm x 0.9 mm Tube voltage: 120 kVp Scan mode: Helical Pitch: 0.938 Slice thickness: 2.0 mm Bowtie Filter: Large Recon kernel: FC18 Tube current: SURE EXPOSURE^a^
CBCT	On‐Board Imager, Varian Medical Systems	Resolution: 0.9 mm × 0.9 mm Tube voltage: 120 kVp Tube current: 80 mA Slice thickness: 2.0 mm Projections: 900 360˚ scan

Abbreviations: CBCT, cone‐beam computed tomography; CT, computed tomography; MRI, magnetic resonance.

^a^ SURE EXPOSURE settings: SD 10.0, SureIQ STD.Axial, image thickness 5.0 mm.

**FIGURE 1:**
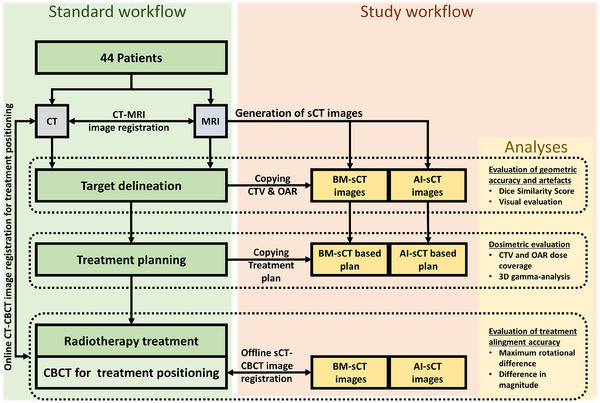
Flowchart of the standard clinical workflow and study workflow.

After acquisition, sCT images were generated with bulk density method,^15^, ^16^ and AI‐based method.^17^ Bulk density method is commercially available and was conducted using the software syngo.via RT Image Suite VB50 (Siemens Healthineers), the AI‐based method was implemented by using a software prototype provided by Siemens Healthineers. For the use of prototype in‐phase and out‐of‐phase DIXON‐MRI images were used for the reconstruction of sCT images. The study workflow is shown in Figure 1.

The manufacturer suggests using a simplified Houndsfield unit (HU)‐to‐electron density curve in dose calculations. However, with our dataset, the sCT algorithms produced higher HU‐values than in the range of the curve provided by the manufacturer. Therefore, we used same HU‐to‐electron density calibration curve for sCT dose calculations as with our TPCT, which covered the full HU‐range of the sCT images.

### 2.3 Geometric accuracy and artifact evaluation

In the standard protocol, rigid co‐registration of MRI and CT images are conducted by medical physicist and the validity of the co‐registration is verified by oncologist prior the delineations of clinical target volumes (CTV) and organs at risk (OAR). The CTVs and OARs (in this study bladder and rectum) were transferred from the original CT to the generated sCT images. During this step, each sCT image was visually evaluated for artifacts, alongside with corresponding MRI and CT images of the patient. According to the quality assurance, the MRI used in this study had distortion less than 1 mm at 200 mm and less than 2 mm at 400 mm from the isocenter (GRADE phantom, Spectronic Medical) scans which is in line within the national recommendations for MRI‐only.^18^ Thus, the major inaccuracies in the image geometry stem from errors in patient geometry (i.e., interscan patient position variability and motion during MRI).

For evaluating the geometric accuracy, body and bone contours of sCT and CT images were compared with dice similarity score (DSC). Body contours and bone contours were generated with automated tools of the contouring‐workspace in the treatment planning system (Eclipse v.13.6, Varian Medical Systems). The body contour was generated by thresholding at ‐350 HU and cropping it alongside *z*‐axis (craniocaudal‐axis), because the imaging field of view (FOV) in CT and MRI were different. The superior part of the body contour was set to the intersection of the lumbar vertebrae and sacrum (L5‐S1 joint) and the inferior part of the body contour was set to the most inferior part of ischium. Automated bone segmentation to generate the bone contour was applied with same volume of interest as body contour.

### 2.4 Dosimetric evaluation

The treatment planning system used in this study was Eclipse v 13.6 (Varian Medical Systems). All treatment plans were Volumetric Modulated Arc Therapy (VMAT) with 6 MV energy and with 2 arcs. Original plans were optimized and calculated with an Anisotropic Analytical Algorithm (v. 13.6.23) with 2.5 × 2.5 × 2.5 mm^3^ calculation grid. Plans consisted of SIB, sequential, and no boost plans (see Table 1) but only the highest dose prescription area (prostate or prostate bed) was included in this study for further evaluation. The plans were copied to the sCT images and recalculated with same calculation algorithm and calculation grid as the original plans and with HU‐electron density curve as treatment planning CT (TPCT). No optimization, normalization or changes in monitor units (MU) were done.

For dosimetric evaluation, CTV and OAR dose coverages (D_98%_, D_50%_, D_2%_, and D_max_) were determined from a dose‐volume histogram (DVH). The dose values presented in this study are relative to the prescribed dose. Furthermore, global 3D gamma‐analysis was conducted with Verisoft analysis software (v 7.2.0.68 (PTW, Freiburg GmbH)). The dose distribution from the TPCT‐based plan was used as a reference dataset in the gamma‐analysis and dose distributions from the sCT‐based plans were used as the evaluation datasets. Multiple gamma index (1mm1% and 2mm2%) criteria were used in calculating the passing rates of the gamma‐analysis with the following settings: gamma‐index ≤1.0 (global max), low‐cut dose <10%. This range of gamma index criteria corresponds to the variety of the criteria used in the previous literature.^1^

### 2.5 Treatment positioning accuracy

Rigid co‐registration between CBCT and sCT images was conducted similarly as in the treatment machine during the treatment positioning. From the middle of the treatment course, one CBCT image set per patient was co‐registered with sCT images. The co‐registration was conducted in 6 DOF or in 4 DOF (*x*, *y*, *z* translations, and *x*‐*y*‐plane rotation, or in Varian terminology; VRT, LAT, LNG, and ROT), depending on whether the original treatment positioning was conducted with a treatment system that had 6DOF‐ or 4DOF‐table. In standard workflow, fiducial markers or surgical clips are used in treatment positioning. If fiducial markers or clips are absent, the positioning is done prostate‐centered based on the soft tissue and bony structures. As the sCT algorithms did not generate these markers the co‐registration between CBCT and sCT images was conducted in this manner.

For evaluating the treatment positioning accuracy, displacements between CT‐CBCT and sCT‐CBCT image registrations were calculated in three translational and three rotational directions. Furthermore, magnitude M of the translational directions was calculated (see Equation 1) to evaluate the sum effect of the displacements. Equation (1), magnitude *M*:

(1) $$M=\sqrt{X^{2}+Y^{2}+Z^{2}},$$

where *X*, *Y*, and *Z* are translational shifts alongside in *X*‐, *Y*‐, and *Z*‐axis.

### 2.6 Statistical analysis

Statistical analyses were conducted for the patient data (n = 44) to evaluate whether AI‐based sCT produces significantly different results from BM‐based sCT. The normality of the datasets was tested with Kolmogorov‐Smirnov and Shapiro‐Wilk tests. As the datasets were not following normal distribution, a pair‐wise non‐parametric test, Wilcoxon signed rank test, was selected to be used in comparisons between AI‐sCT and BM‐sCT. The parameters which were compared were geometric accuracy (DSC of Bone and Body contours), the treatment position accuracy (co‐registration difference magnitude and maximum degree), and the parameters from dosimetric analyses, which consist of DVH parameters (D_98%_, D_50%_, D_2%_, and D_max_ values of CTV, Rectum and Bladder) and gamma‐analysis values (1mm1% and 2mm2%).

To provide insight whether the observed differences have clinical relevance, the parametric results were transformed into dichotomous datasets by evaluating each parameter with pass/fail criteria against reference data (data provided based on TPCT) and conducting the McNemar's test. The sCT algorithms producing dose differences <2% have been suggested to be clinically acceptable.^2^ Therefore the passing limit for DVH parameters(D_98%_, D_50%_, D_2%_, and D_max_ values of CTV, Rectum, and Bladder) was set to the parameter being less than 2% different from the corresponding DVH parameters obtained from data‐based on TPCT. For the gamma‐analysis results, the passing rate values higher than 90%, 95%, and 98% of the gamma‐analysis were set as passing limits, representing the variation of used limits found in literature,^1^ or used in routine quality assurance in our local clinics. Each passing rate limit was evaluated separately. The geometric accuracy evaluation for the bone DSC had issues due to differences in patient positioning between MRI and CT scans. Thus, two different passing limits for DSC evaluations were used. The passing criteria of higher than 0.98 were set for the body contour. The results between the bone contours were well below this limit in all cases due to the variation in patient positions, more specifically in the position of femurs, between MRI and CT scans. Therefore, we set lower passing criteria of 0.7 for the bone contour DSC. All statistical analyses were conducted using IBM SPSS Statistics‐software (v.29.0.1.0).

## 3 RESULTS

### 3.1 Geometric accuracy and artifact evaluation

**FIGURE 2:**
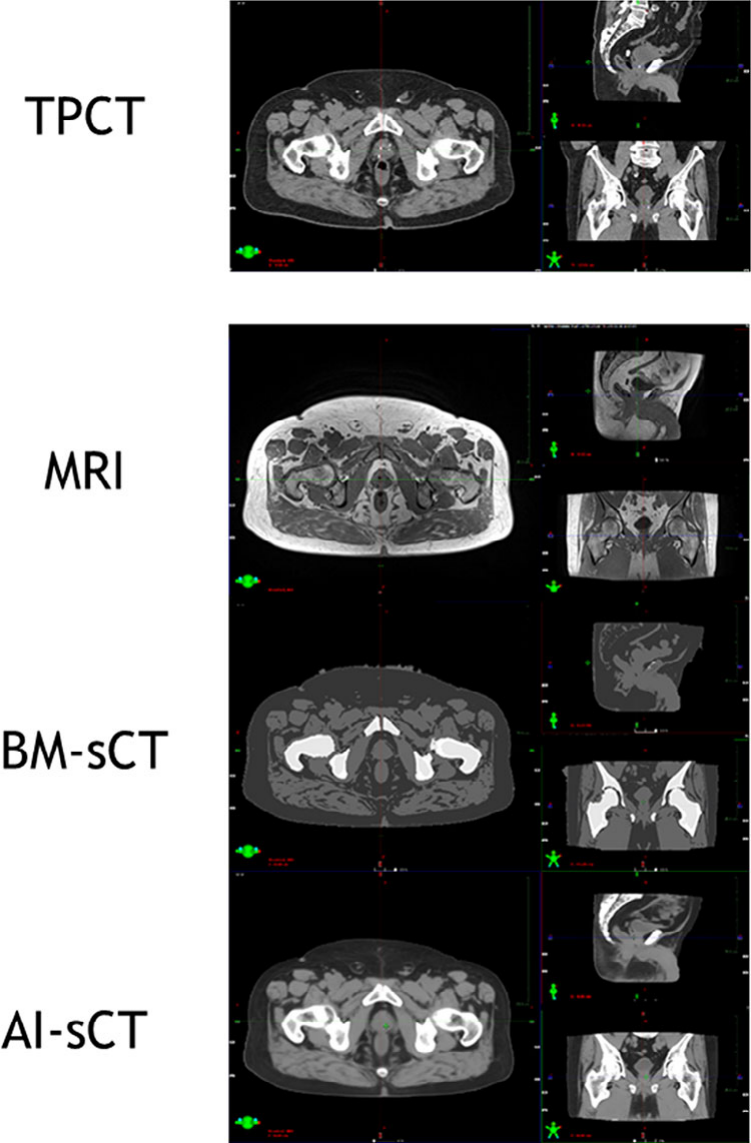
Example case of sCT algorithms on pelvis area. From the top images of treatment planning CT, MRI, BM‐ sCT, and AI‐ sCT. AI, artificial intelligence; BM, bulk‐density‐method; CT, computed tomography; MRI, magnetic resonance; sCT, synthetic computed tomography.

**FIGURE 3:**
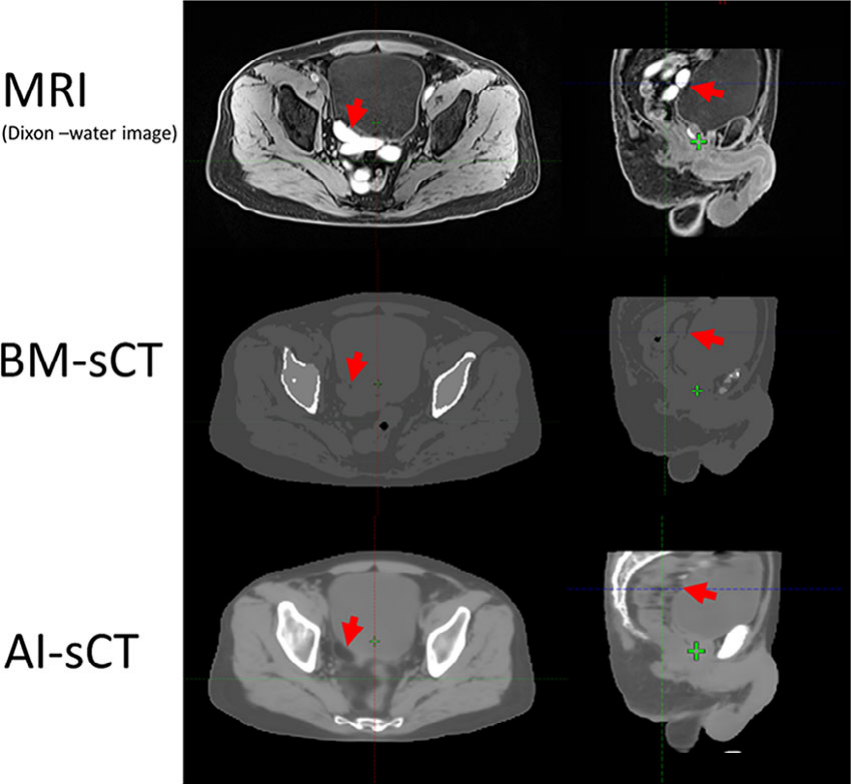
Example case of MRI sequence artifact causing missing bowel loops in AI‐sCT. Red arrows indicate corresponding bowel loops which show also a high‐intensity signal in the MRI Dixon‐water image, as soft tissue indistinguishable from the bladder wall in BM‐sCT and as visceral fat in AI‐sCT. AI, artificial intelligence; BM, bulk‐density‐method; CT, computed tomography; MRI, magnetic resonance; sCT, synthetic computed tomography.

The major observations from visual inspection of the sCT images were related to how sCT algorithms handled fiducial markers, the air in bowels, and apparently MRI imaging‐related artifacts. The fiducial markers were generated as soft tissue by both sCT algorithms (see Figure 2). The air in the patient's bowels was set as air in BM‐sCT while AI‐sCT tried to generate the air in the bowels as soft tissue at the expense of bowel morphology. Furthermore, some inaccuracies in bowels soft tissue classification were observed which seemed to occur with patients also having high‐intensity signals in the bowels in the Dixon water image (see Figure 3). Geometric inaccuracy was not observed inside the body contour in sCT images. However, slight mismatches in patient positioning were observed between MRI and CT which can be seen also in subtraction images between sCT and CT (see Supplementary Material).

**FIGURE 4:**
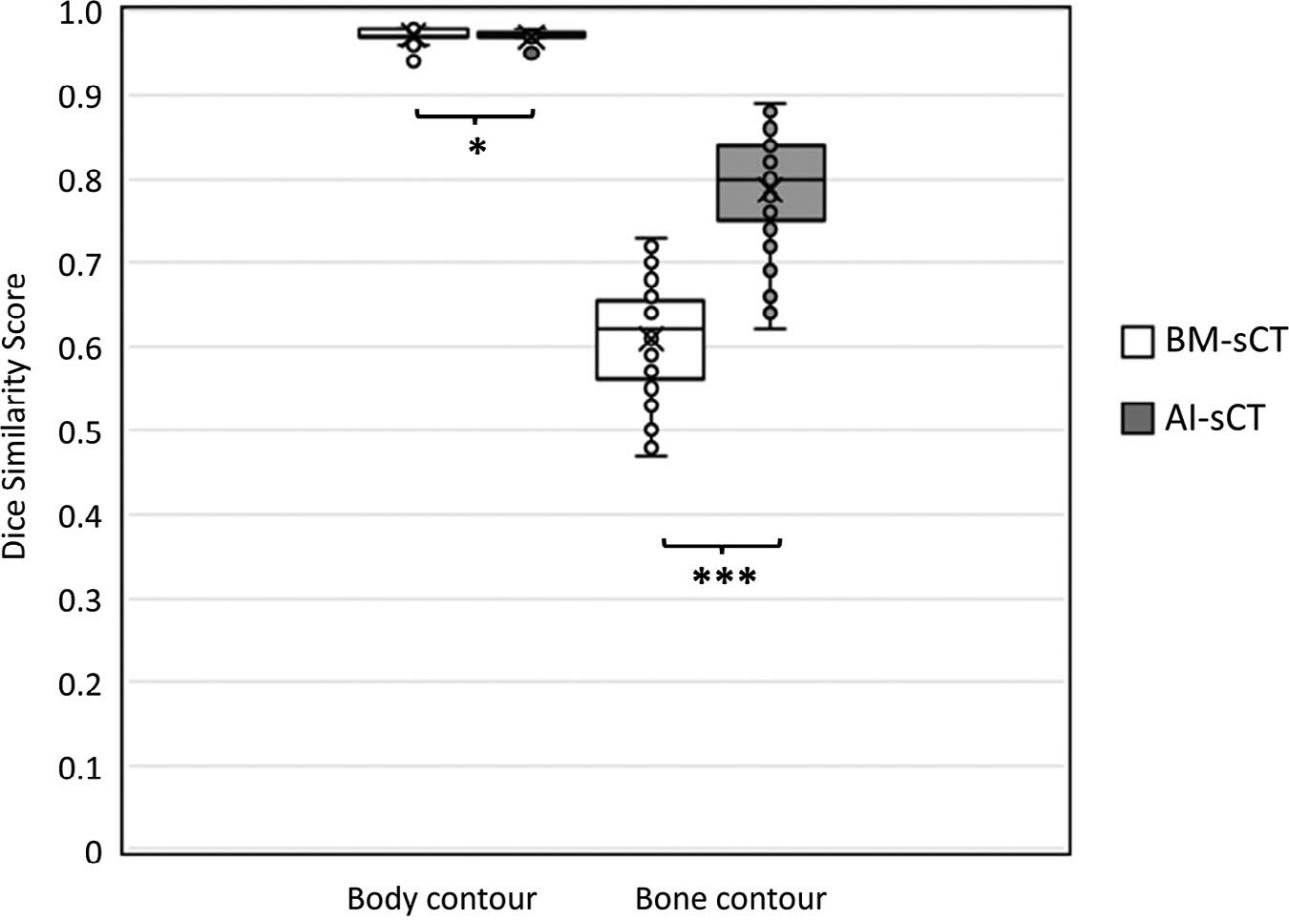
Dice similarity scores of body and bone contours between sCT and CT images. Asterisks (*) indicate statistically significant (***: *p* <0.001, *: *p* <0.05) difference between BM‐sCT and AI‐sCT by Wilcoxon signed rank test. AI, artificial intelligence; BM, bulk‐density‐method; CT, computed tomography; sCT, synthetic computed tomography.

The DSC of the body and bone contour is shown in Figure 4. Both BM‐sCT and AI‐sCT showed similarly good results when body contours were compared with CT as DSC ranged from 0.96–0.98. When comparing bone contours, the DSC for BM‐sCT and CT was in the range 0.47–0.73 while in comparison between AI‐sCT and CT, the range was 0.62–0.89. The difference between AI‐sCT and BM‐sCT was statistically significant *p* = 0.042 and *p* <0.001 for body and bone contour DSC analyses, respectively. The McNemar's test, with passing criteria DSC > 0.98 for body contours and DSC >0.7 for bone contours, showed a statistically significant (*p* <0.001) difference between AI‐ and BM‐sCT in bone contour comparison but not in body contour comparison.

### 3.2 Dosimetric evaluation

**FIGURE 5:**
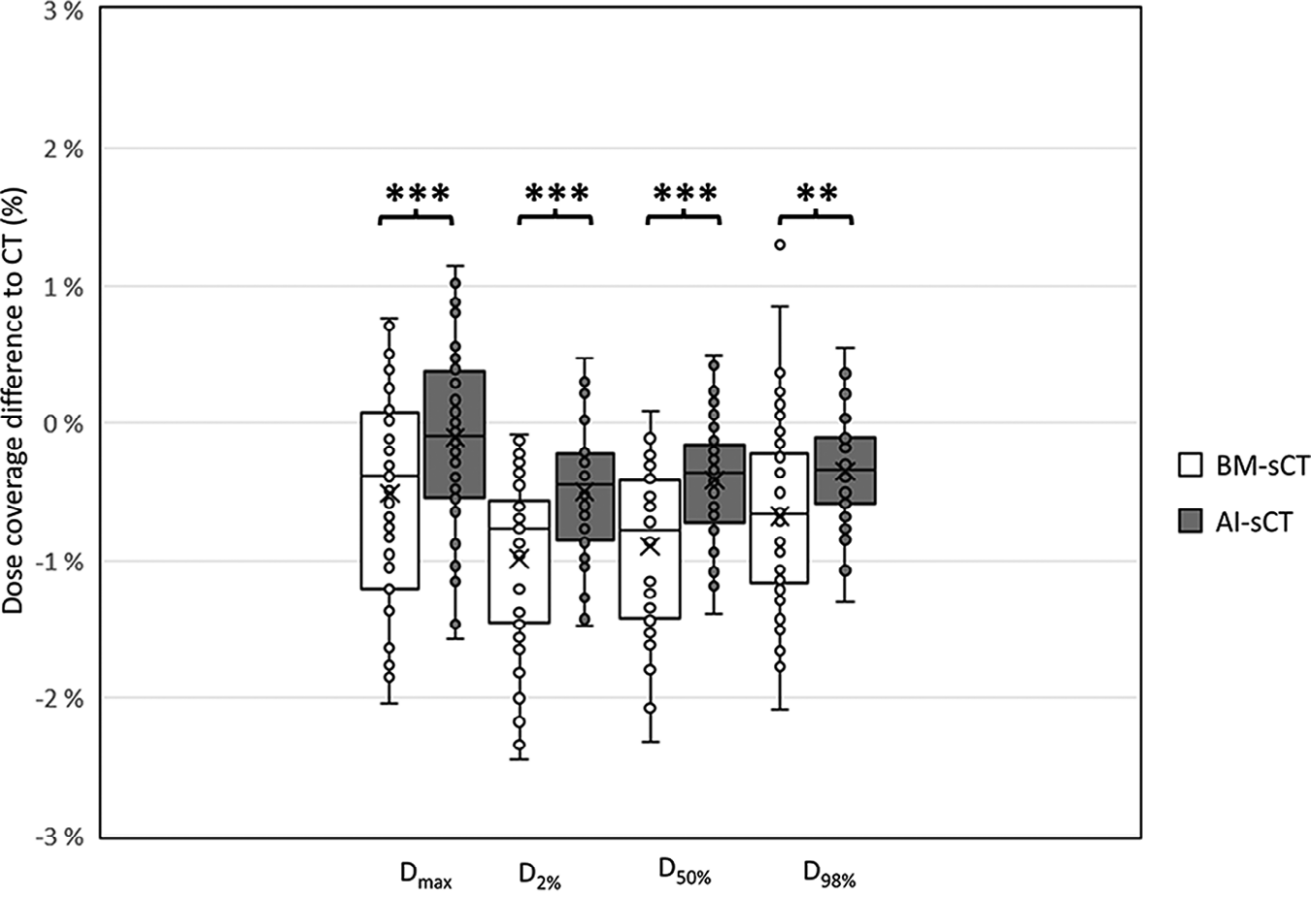
Dose coverage differences of CTV between CT and sCTs. Mean (*x*) and median (line in the box) are closer to 0 with AI‐sCT than with BM‐sCT with all dose coverage parameters. Asterisks (*) indicate statistically significant (***: *p* <0.001, **: *p* <0.005) difference between BM‐sCT and AI‐sCT by Wilcoxon signed rank test. AI, artificial intelligence; BM, bulk‐density‐method; CT, computed tomography; CTV, clinical target volumes; sCT, synthetic computed tomography.

Differences in dose coverages of CTV are presented in Figure 5. Comparison between BM‐sCT and CT showed that in 90% of all patients, the difference in CTV (D_2%,_ D_50%_, D_98%_, or D_max_) was less than 2% and in 59% of all patients, the difference was less than 1%. Similarly comparing AI‐sCT to CT 98% of patients the difference in any CTV dose coverage value was less than 2% and 88% of patients had difference less than 1%. The CTV dose coverage between AI‐sCT and BM‐sCT was significantly different (*p* <0.001, *p* <0.001, *p* = 0.003, *p* <0.001) by all CTV's DVHmetrics (D_2%,_ D_50%_, D_98%_, and D_max_, respectively). However, McNemar's test did not show a statistically significant difference between AI‐sCT and BM‐sCT with any of the CTV's DVH metrics.

When comparing dose coverages in OARs the deviation between CT and sCT's was higher than in CTVs (see Supplementary Material). For the rectum, less than 2% difference in dose coverage values was achieved in 83% of patients with BM‐sCT and 88% in patients with AI‐sCT. For the bladder, a 2% or less difference was achieved in 95% of patients with BM‐sCT and 90% in patients with AI‐sCT.

Comparison of AI‐sCT and BM‐sCT produced DVH‐parameters for the bladder and rectum revealed a statistically significant difference, in favor of AI‐sCT, with bladder D_2%_ (*p* <0.001), bladder D_max_ (*p* = 0.002), rectum D_2%_ (*p* = 0.004), and with rectum D_max_ (*p* <0,001). McNemar's test, with passing criteria of less than 2% absolute difference from the reference value (TPCT), showed that AI‐sCT outperformed BM‐sCT with the following OAR DVH‐metrics: bladder D_98%_ (*p* = 0,003), rectum D_98%_ (*p* = 0,031), and rectum D_max_ (*p* = 0,039). However, BM‐sCT was better than AI‐sCT with bladder D_50%_ (*p* <0,001) and rectum D_50%_ (*p* = 0,039).

**FIGURE 6:**
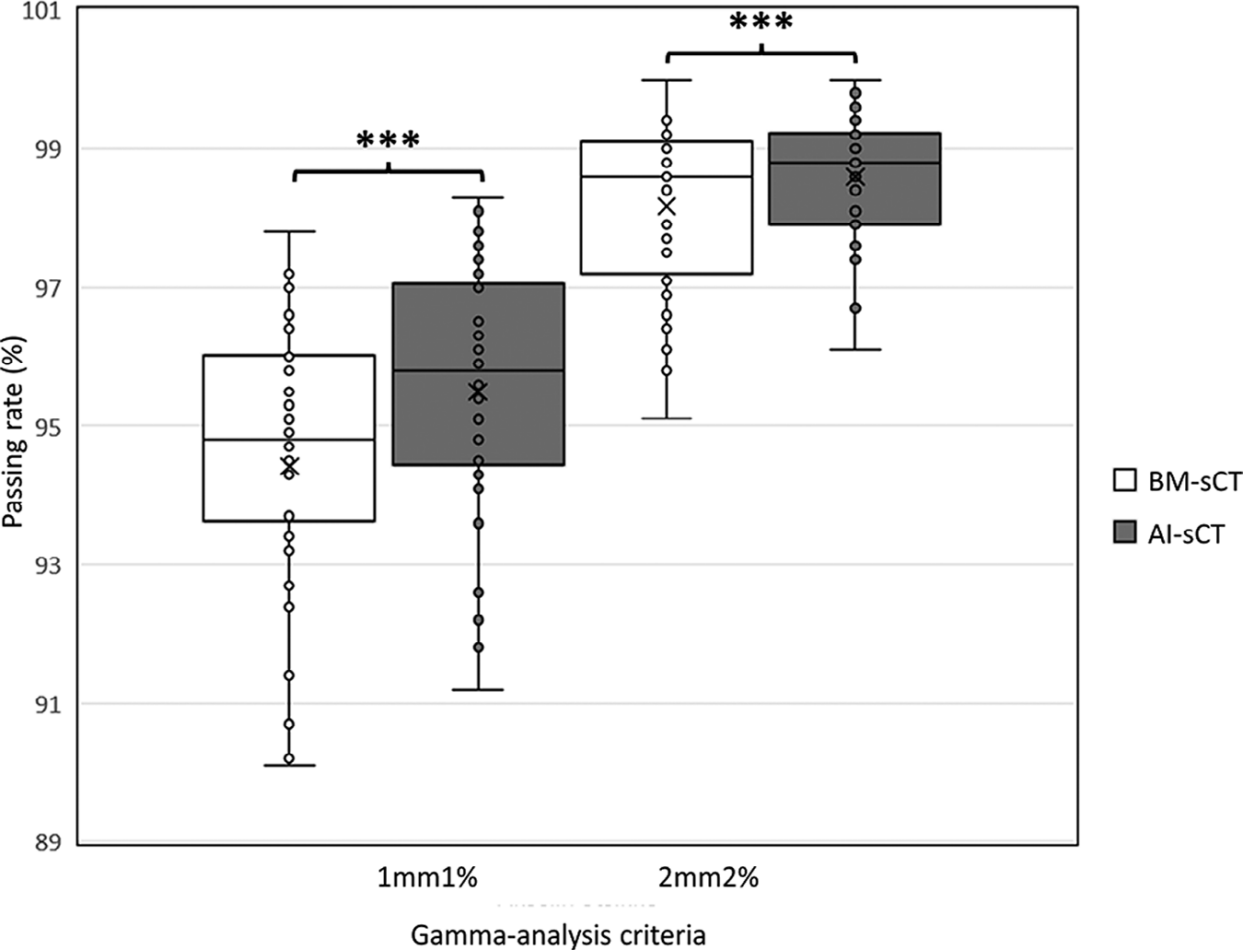
Gamma‐analysis passing rates with 1 mm 1% and 2 mm 2% ‐criteria. Asterisks (*) indicate statistically significant (***: *p* <0.001) difference between BM‐sCT and AI‐sCT by Wilcoxon signed rank test. AI, artificial intelligence; BM, bulk‐density‐method; sCT, synthetic computed tomography.

Gamma‐analysis results are shown in Figure 6. AI‐sCT had a mean passing rate of 95.5% and 98.6%with 1mm1% and 2mm2% criteria, respectively. Similarly, for BM‐sCT the mean passing rates were 93.4% and 98.2%.

The difference between AI‐sCT and BM‐sCT was statistically different with 1mm1% and 2mm2% criteria's (*p* <0.001 and *p* <0.001, respectively). However, according to McNemar's no statistical difference between AI‐sCT and BM‐sCT was observed in gamma‐analysis.

### 3.3 Treatment positioning accuracy

Differences between CBCT‐to‐CT image registration values and CBCT‐to‐sCT image registration values are presented in Table 3. For the boxplot of these values see Figure S3. Statistical analysis showed no statistically significant differences in treatment position accuracy between AI‐sCT and BM‐sCT.

**TABLE 3:** Treatment positioning accuracy.

sCT algorithm	Direction	Mean ± SD	Maximum absolute difference
BM‐sCT	Translational *X*	0.83 ± 0.67 mm	3.1 mm
	Translational *Y*	1.28 ± 1.1 mm	4.2 mm
	Translational *Z*	1.75 ± 1.26 mm	4.4 mm
	Magnitude	1.43 ± 1.20 mm	4.1 mm
	Rotational *X*	1.19 ± 1.09°	4.0°
	Rotational *Y*	0.4 ± 0.42 °	1.8°
	Rotational *Z*	0.77 ± 0.75°	3.9°
AI‐sCT	Translational *X*	1.14 ± 0.93 mm	4.3 mm
	Translational *Y*	1.40 ± 0.93 mm	4.2 mm
	Translational *Z*	1.63 ± 1,23 mm	4.7 mm
	Magnitude	1.57 ± 0.99 mm	3.8 mm
	Rotational *X*	1.13 ± 1.07°	4.0°
	Rotational *Y*	0.43 ± 0.48°	2.4°
	Rotational *Z*	0.78 ± 0.76°	3.9°

*Note*: The sCT‐CBCT co‐registration values were subtracted from CT‐CBCT co‐registration values in 6 DOF. Mean, standard deviation (SD), and maximum difference values are presented in the table.

Abbreviations: CBCT, cone‐beam computed tomography; CT, computed tomography; DOF, degrees‐of‐freedom.

## 4 DISCUSSION

In summary, our results show that the sCT algorithms used in this study are clinically usable and comparable with other algorithms found in the literature. Previous studies from AI‐based sCT algorithms have shown gamma passing rates ranging from > 99% to 91% in the pelvic area.^9^, ^19^, ^20^ Similarly for the bulk method gamma passing rates are ranging from 99% to 90%.^8^, ^9^ Recently published article comparing sCT algorithms in patients with other pelvic treatments (the rectum, anal canal, cervix, or endometrium),^11^ showed similar dose differences in DVH evaluation as in our study with mean CTV, bladder, and rectum differences being less than 2% while bladder and rectum DVH had few outliers with higher dose differences. Interestingly, another comparative study of the same algorithms but based on 1.5T MRI images provided slightly worse results.^4^ Gamma passing rates (1mm1% criteria) for AI‐sCT and BM‐sCT were 91% and 82%, respectively. While in our study the gamma analysis with 1mm1% criteria resulted in higher passing rate values (AI‐sCT:95.5%, BM‐sCT:93.4%). The DVH differences were similar for the treatment area and for the OARs, as in both studies, most of the differences were less than 2% (with some outliers in OARs). Also in both studies, BM‐sCT performed slightly worse in the dose evaluations compared to AI‐sCT. This seems to indicate that sCT performs slightly better for 3T, however, no definitive conclusion can not be made as the patient data between these studies is different.

From the sCT algorithms studied here, AI‐based sCT provided closer match to TPCT almost with all parameters used in dosimetric evaluation (DVH parameters: CTV (D_2%,_ D_50%_, D_98%_, D_max_), bladder(D_2%_, D_max_), rectum(D_2%_, D_max_), and gamma analysis). Furthermore, the difference between BM‐sCT and AI‐sCT was statistically significant with these parameters. However, McNemar's test, AI‐sCT showed significantly better results only with bladder(D_98%_), and rectum(D_98%_, D_max_). In addition, according to McNemar's test, BM‐sCT outperformed AI‐sCT with DVH‐parameters bladder(D_50%_) and bladder(D_50%_). The results of this study show that eventhough AI‐based sCT produces closer dosimetric equivalence to TPCT‐based calculation, BM‐based sCT produces also clinically acceptable results. In this study, the pass‐fail criterias to McNemar's test were selected by our clinic's standards. The results of McNemar's test are heavily dependent on the selection of the pass‐fail criteria. Thus lower or higher passing criteria, based on legislative standards, international or national recommendations, or even on clinic's own standards, need to be evaluated before interpreting dosimetric results of this study.

The treatment positioning accuracy was similar with both tested algorithms (see Table 3). The difference to TPCT‐based positioning was low and as visual evaluation confirmed that dose distribution with sCT‐based treatment positioning would fulfill ICRU criteria,^21^ it seems that the treatment position accuracy with both sCT methods is clinically acceptable. In literature, mean differences in different translational axes are reported ranging from 3.5 mm to less than 0.5 mm.^7^, ^9^, ^22^, ^23^ However, in our study we have reported the absolute mean difference (i.e. mean of the absolute values of the differences) in Table 3 to better present the average errors in treatment positioning accuracy. Previous studies are showing higher outliers and standard deviations^7^, ^9^, ^22^, ^23^ in both negative and positive directions indicating that the absolute mean difference could be higher than in our study. Unfortunately, in a previous study comparing AI‐sCT and BM‐sCT with 1.5T MRI, researchers did not conduct treatment positioning accuracy testing, thus the positioning performance of these algorithms with 1.5T remains to be elucidated.^4^ In our study, we found no statistically significant differences in treatment positioning accuracy between AI‐sCT and BM‐sCT. Furthermore, in our study, the mean of absolute difference (ranging from 0.83 to 1.75 mm, see Table 3) is in the order of the voxel resolution of the MRI (1.2 mm × 1.2 mm × 1.2 mm) making the difference clinically negligible.

The differences in patient positioning and internal anatomy between the different imaging modalities are a major error source. This limitation cannot be avoided in these types of multimodal studies but it has to be addressed. In this study, errors were minimized by limiting the volume of interest for the analysis and if necessary excluding patients with major differences in patient positioning, or with patients with extensive differences in internal anatomy (i.e. due to large air pockets in bowels) between the MRI and CT. While the DSC of the body contour was good, 0.97 on average with both sCT methods, the DSC in bone contour was worse with both sCT images indicating differences in bony structures. The main discrepancies were in the positioning of the femurs. The “sliding HU‐values” also provide a better presentation of bony structures in the AI‐sCT, which explains the significantly higher DSC of AI‐sCT when compared to BM‐sCT. In comparison studies found in literature, the AI‐sCT DSC results of body and bone contour range between 0.99–0.98 and 0.94–0.85, respectively.^19^ Furthermore, Yoo et al.^19^ state similar issues of inter‐scan variation between CT and MRI resulting in relatively poor DSC values.

The visual evaluation revealed that fiducial markers are not generated by either of the sCT algorithms. In our clinic, we use triggered imaging for hypofractionated prostate treatments. This means that to implement MRonly protocol for this patient group, fiducial markers need to be defined from other MRI images, that is, Dixon water images. The visual evaluation also revealed that prescreening of MRI images before subjecting the patients to MRonly protocol might be needed as movement artifacts and air in bowels would produce errors or even prevent bowels segmentation from generated sCT images. Furthermore, AI‐sCT produced a few cases in which the bowel loops were generated as visceral fat. With further investigation, these cases had high‐intensity signals in the bowel loops in the Dixon‐water images. Even though the AI‐sCT algorithm only utilizes in‐phase and opposing‐phase images. As the core reason for this artifact remains to be elucidated, the spatial location of this artifact could be roughly evaluated from Dixon‐water images prior sCT generation.

In addition to the pelvic area, the BM‐sCT and AI‐sCT have been shown to provide clinically feasible results in the brain region.^2^, ^5^, ^9^, ^10^ The BM‐sCT methods have been reported to have dose discrepancies > 2% due to the homogeneity of the HU‐values,^2^ which is not an issue with AI‐sCT algorithms. Furthermore, AI‐sCT algorithms show promising results for high‐precision techniques such as stereotactic brain radiotherapy.^10^ This suggests that in the future sCT development is mainly focused on the development of AI‐sCT algorithms in other treatment areas.

In summary, the AI‐based sCT algorithms will provide higher accuracy compared to the BM‐sCT also with 3T MRI. 3T MR‐only is feasible for specific prostate patient demographics; however, utilization requires knowledge of the limitations of these algorithms. The quality of AI‐based algorithms depends on their training sets and in abnormal cases, the algorithms might produce artifacts on their own. Based on our experience the AI‐based algorithm used in this study would work perfectly for patients without atypical anatomy or without implants (including fiducial markers), and with no large air pockets in the bowels. After the careful patient selection, personnel who are familiar with the limitations of the sCT‐algorithms should review the sCT images together with MRI to prescreen potential artifacts. This study provided valuable information to our clinic and it was conducted as part of the clinical commissioning of the MR‐only protocol in addition to the oncologist evaluation of the sCT images. We recommend clinical commissioning of the sCT algorithms, with a sufficient amount of patients before utilizing them with different patient groups.

## 5 CONCLUSIONS

Based on our results, we can conclude that both synthetic computed tomography algorithms tested in this study are clinically usable with most of the patients going through prostate 3T MR‐only workflow. While AI‐based algorithm out‐performed the bulk density algorithm in 3T, in a few cases it generated artifacts in the bowels. Furthermore, the lack of fiducial markers from sCT images can increase errors in treatment alignment. We recommend commissioning of sCT algorithms with retrospective analyses from patient data, to reveal the pit‐falls of the algorithms before implementing sCT into the routine workflow.

## AUTHOR CONTRIBUTIONS

All authors took part in the conceptualization, methodology, and writing (review and editing) of the manuscript. Sakari S. Karhula, Piia Karppinen, and Henna Hietala took part in formal analysis, investigation, and data curation. Sakari S. Karhula was responsible for the original draft preparation and visualization. Supervision, Juha Nikkinen; project administration, Sakari S. Karhula, and Juha Nikkinen. All authors have read and agreed to the published version of the manuscript.

## CONFLICT OF INTEREST STATEMENT

The authors declare no conflicts of interest.

## INSTITUTIONAL REVIEW BOARD STATEMENT

“The study was conducted in accordance with the Declaration of Helsinki, and approved by the Institutional Review Board of Ostrobothnia Hospital District (protocol code 60/2020, 9th of April 2020).” for studies involving humans.

## INFORMED CONSENT STATEMENT

Patient consent was waived because this was a retrospective study with data being handled anonymously. The research was conducted with the approval of the Ostrobothnia Hospital District.

## Supporting information



Supplementary Information

## Data Availability

Ethics approval for this study does not allow for the sharing of individual patient scans. Other study data, which does not include patient data sets, are available from the corresponding author on reasonable request.
