# Supplementary material for: Evaluation and comparison of synthetic computed tomography algorithms with 3T MRI for prostate radiotherapy: AI‐based versus bulk density method
Source: J Appl Clin Med Phys. 2024 Nov 29;26(3):e14581. doi: 10.1002/acm2.14581 (PMC11905239; doi:10.1002/acm2.14581)
Supplement: Supplementary file 1 — Supplementary Information [file ACM2-26-e14581-s001.docx]

**Supplementary material**


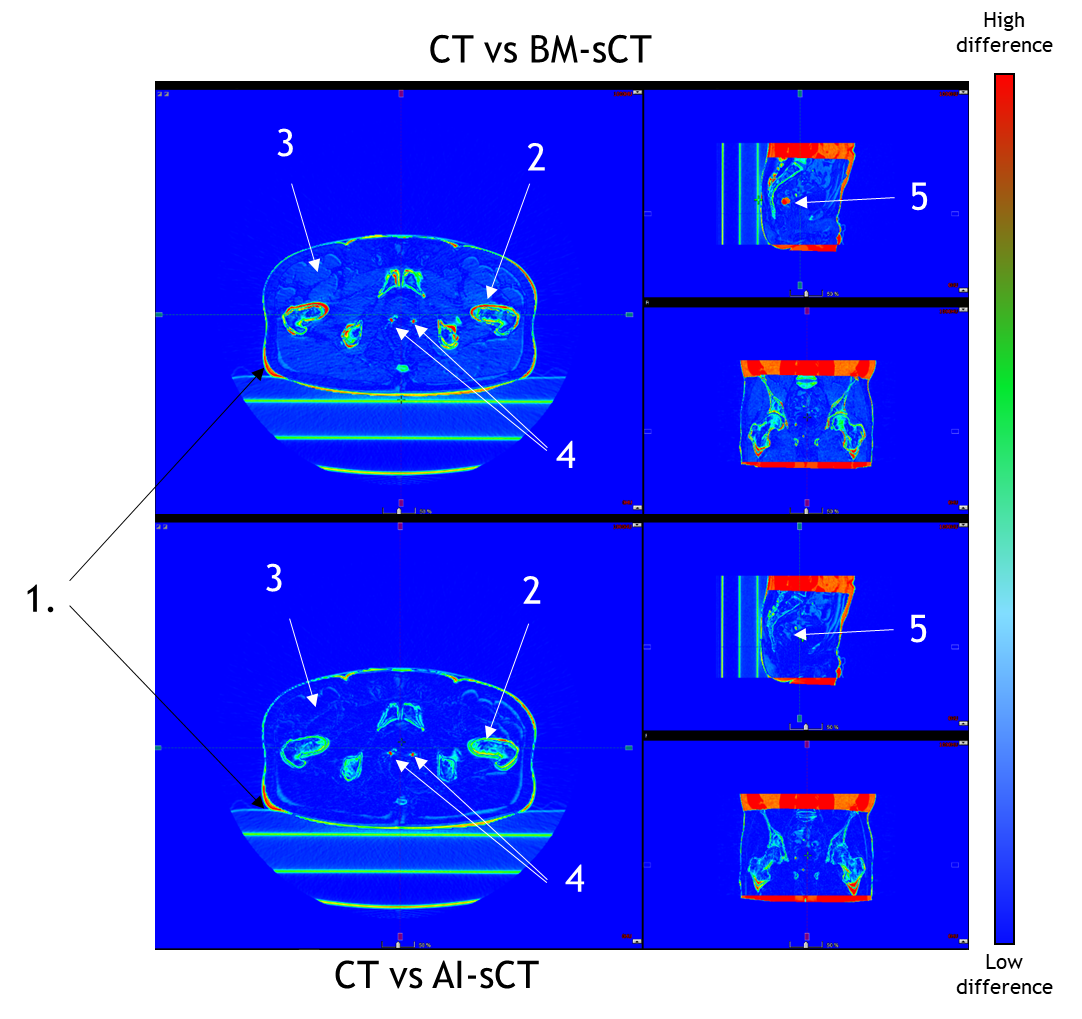


**Supplementary Figure 1.** Example of observations in visual evaluation. On the top subtraction image between CT and BM-sCT on the bottom subtraction image between CT and AI-sCT. The colormap is shown on the right. The observations: 1) No visible geometric artefacts on body contour area. Only geometric artefacts were noticed on the very edges of the FOV. This difference is due difference in patient positioning. 2) Similarly in bones the patient repositioning was seen, especially on femurs. However, slight difference in HU values was systematically observed. 3. When comparing soft tissues, AI-sCT was clearly superior, it presented HU values at much higher accuracy (due the “sliding-HU-property”). 4. Fiducial markers were missing from the sCT images. 5. In few of the cases, patients had air in the bowel. BM-sCT categorized these areas as air but AI-sCT tried to set these as soft tissue in expense of bowel morphology.


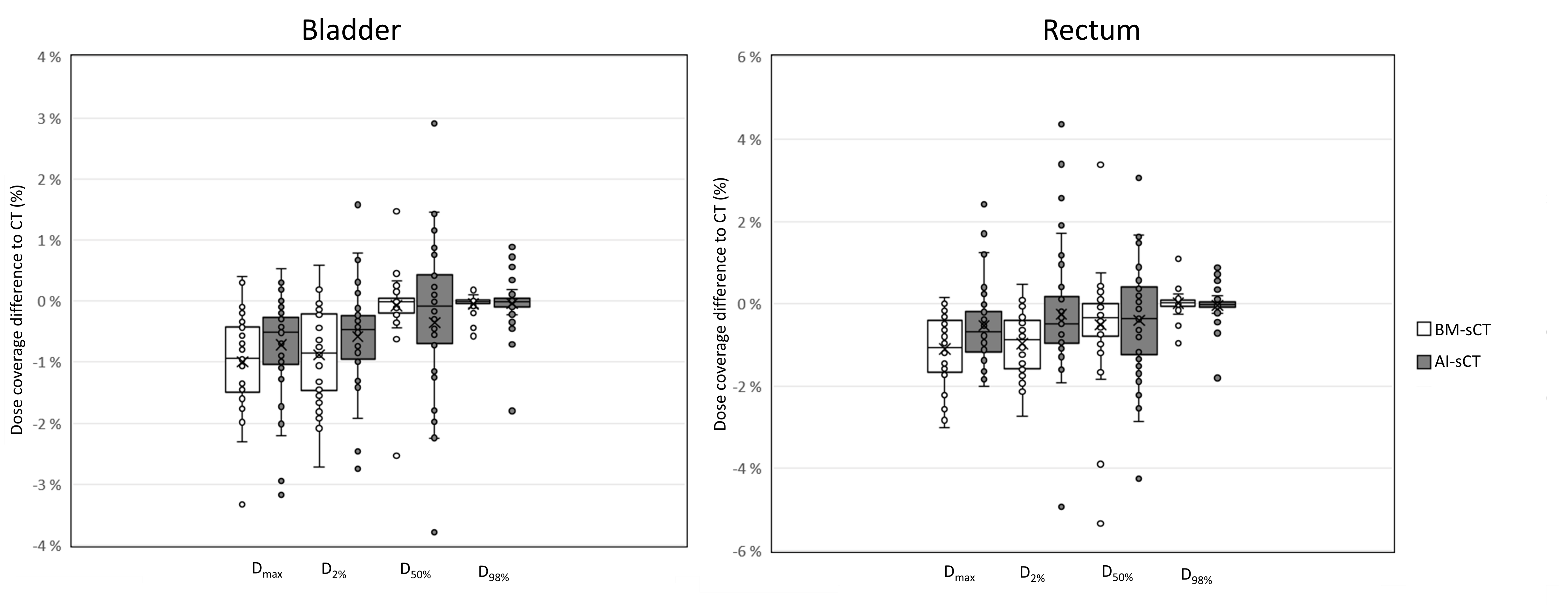


**Supplementary Figure 2.** Dose coverage differences of Bladder and Rectum. In box-plots, mean value is marked as “X” while median is presented as a line in the box.


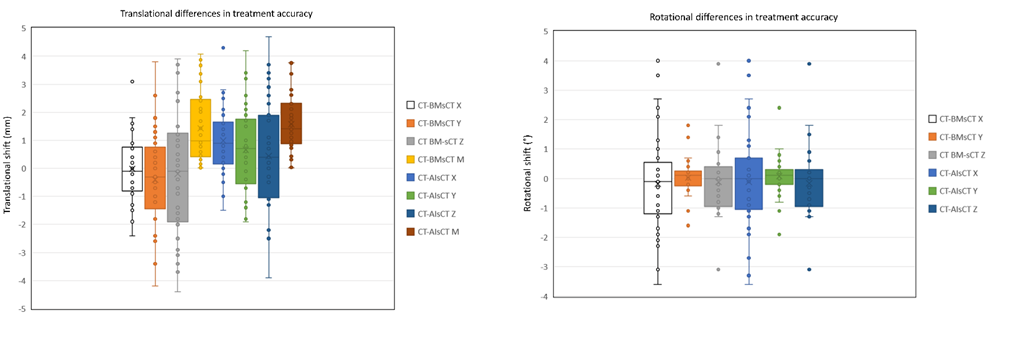


**Supplementary Figure 3.** Treatment positioning accuracy data. On the left, translational differences (in mm) between CT and BM-sCT, and between CT and AI-sCT. Letters X, Y, and Z are the translational and rotational axis, Letter M indicates the calculated magnitude from the translational shifts.
